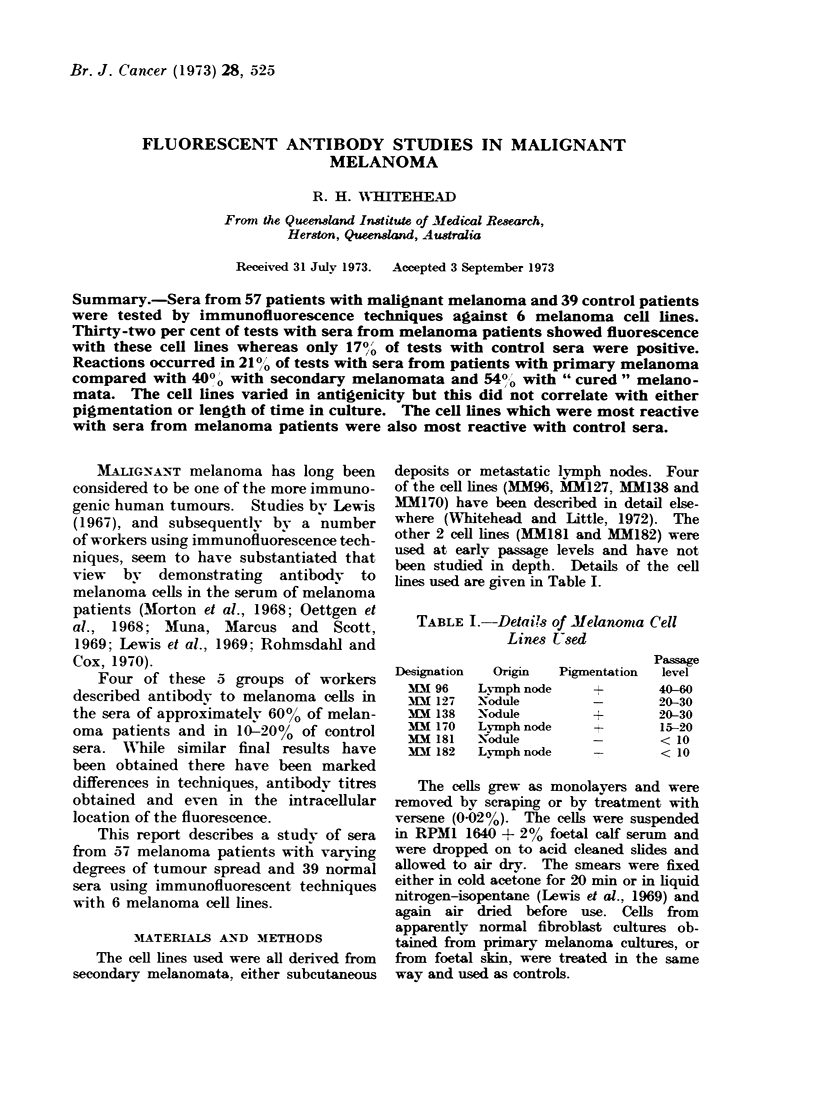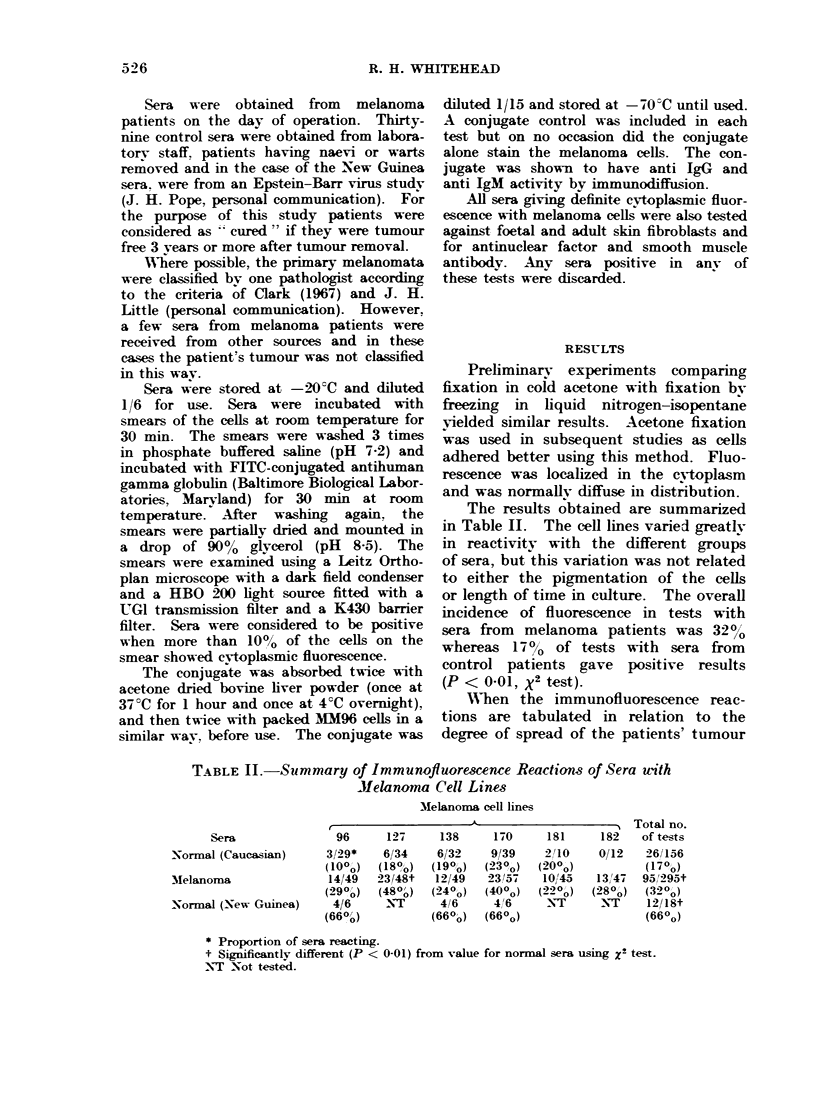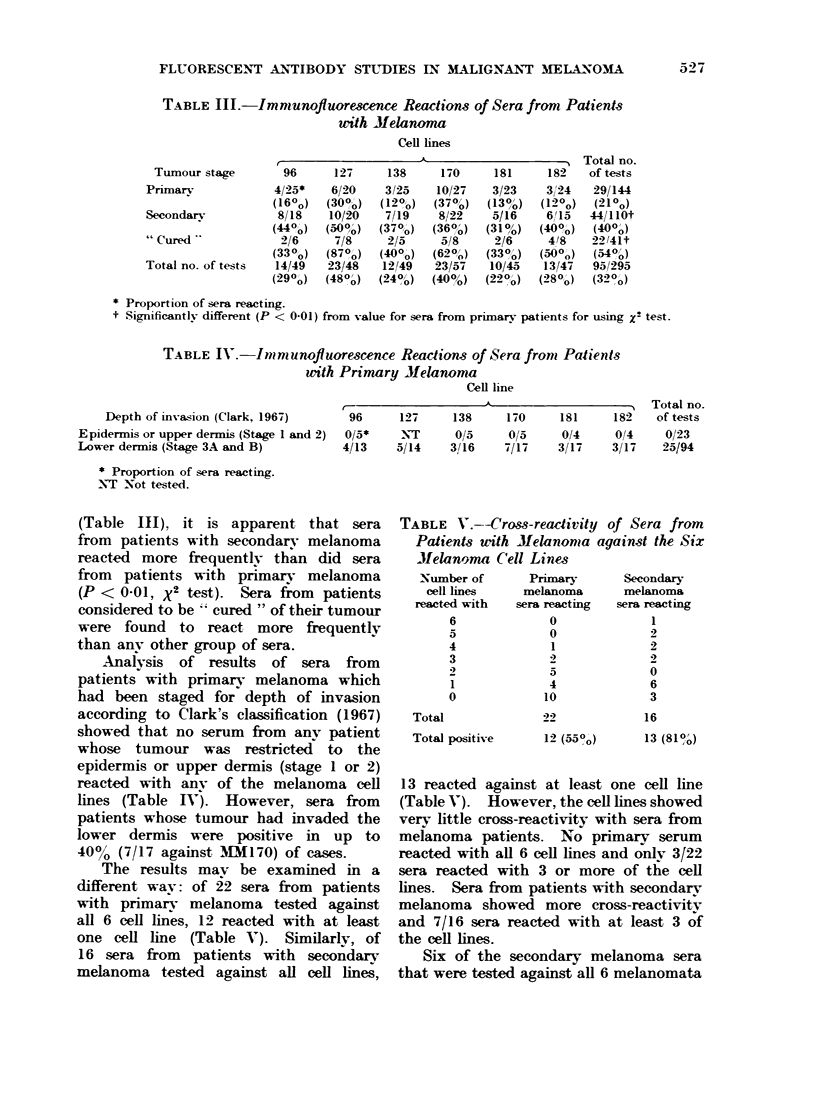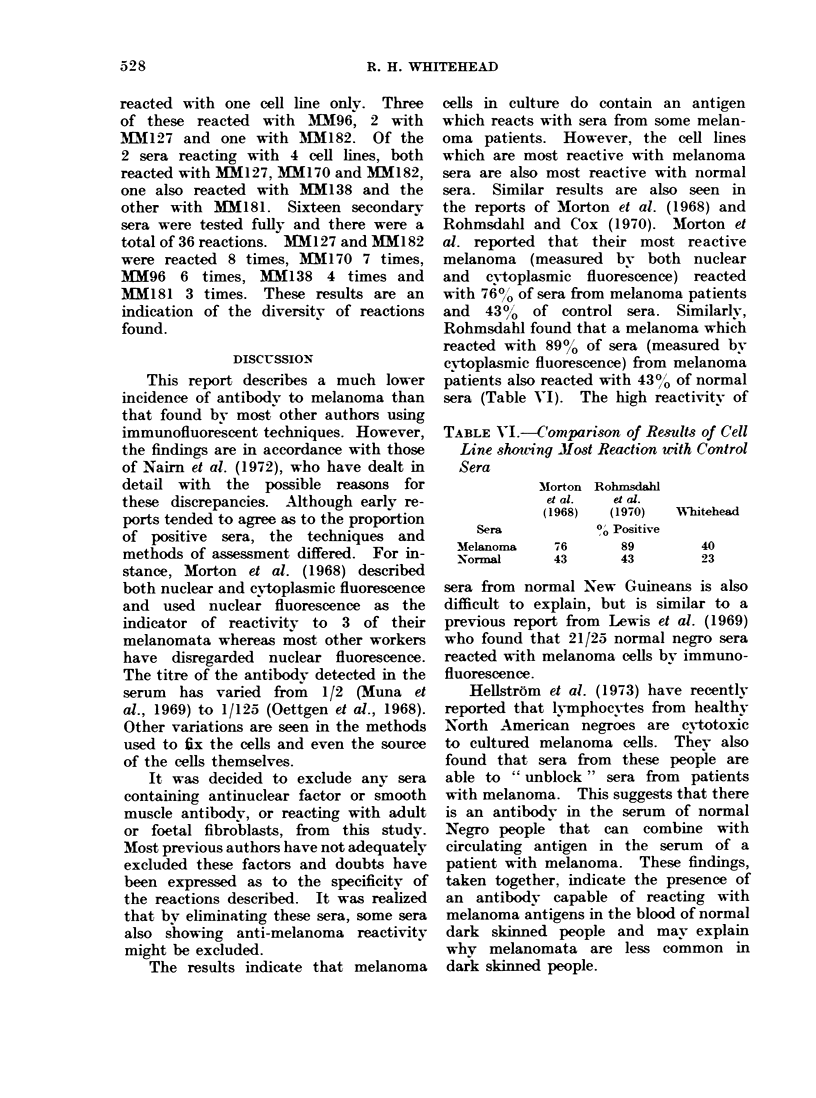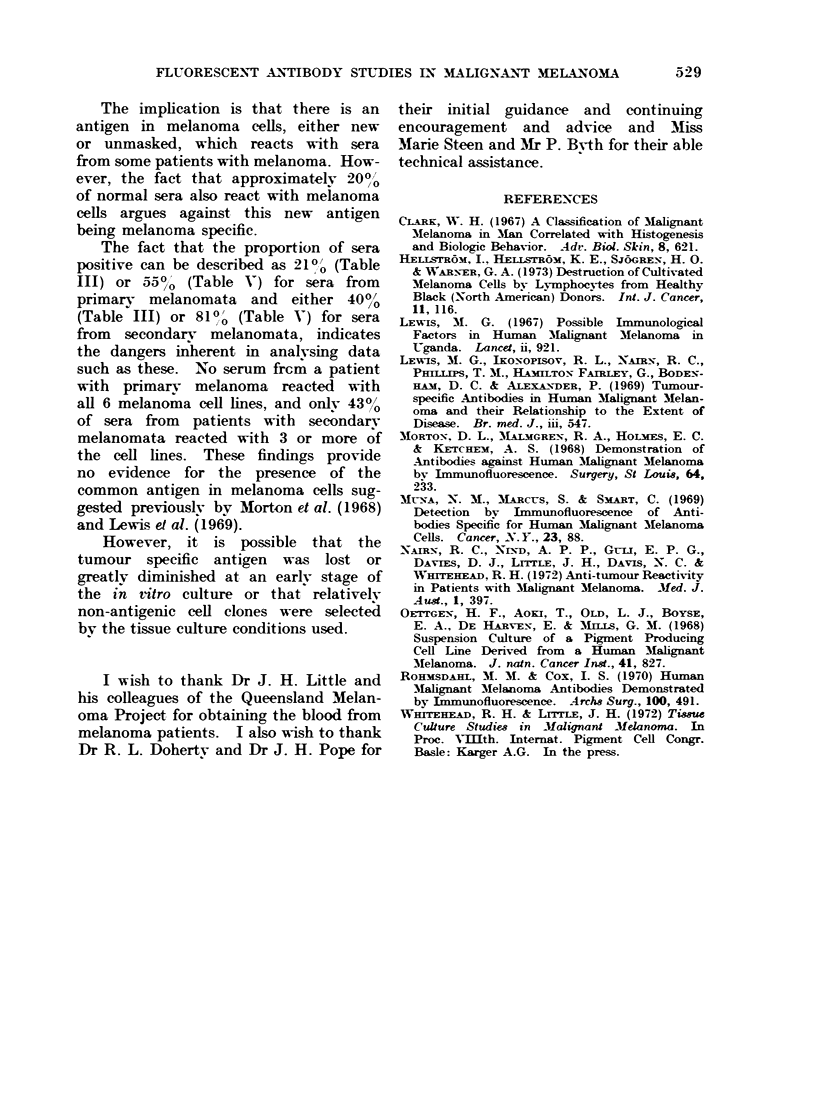# Fluorescent Antibody Studies in Malignant Melanoma

**DOI:** 10.1038/bjc.1973.182

**Published:** 1973-12

**Authors:** R. H. Whitehead

## Abstract

Sera from 57 patients with malignant melanoma and 39 control patients were tested by immunofluorescence techniques against 6 melanoma cell lines. Thirty-two per cent of tests with sera from melanoma patients showed fluorescence with these cell lines whereas only 17% of tests with control sera were positive. Reactions occurred in 21% of tests with sera from patients with primary melanoma compared with 40% with secondary melanomata and 54% with “cured” melanomata. The cell lines varied in antigenicity but this did not correlate with either pigmentation or length of time in culture. The cell lines which were most reactive with sera from melanoma patients were also most reactive with control sera.


*Br. J. Cancer* (1973) **28**, 525

### FLUORESCENT ANTIBODY STUDIES IN MALIGNANT MELANOMA

R. H. WHITEHEAD

*From the Queensland Institute of Medical Research, Herston, Queensland, Australia*

Received 31 July 1973. Accepted 3 September 1973

**Summary.—Sera from 57 patients with malignant melanoma and 39 control patients were tested by immunofluorescence techniques against 6 melanoma cell lines. Thirty-two per cent of tests with sera from melanoma patients showed fluorescence with these cell lines whereas only 17% of tests with control sera were positive. Reactions occurred in 21% of tests with sera from patients with primary melanoma compared with 40% with secondary melanomata and 54% with " cured " melanomata. The cell lines varied in antigenicity but this did not correlate with either pigmentation or length of time in culture. The cell lines which were most reactive with sera from melanoma patients were also most reactive with control sera.**

MALIGNANT melanoma has long been considered to be one of the more immunogenic human tumours. Studies by Lewis (1967), and subsequently by a number of workers using immunofluorescence techniques, seem to have substantiated that view by demonstrating antibody to melanoma cells in the serum of melanoma patients (Morton *et al.*, 1968; Oettgen *et al.*, 1968; Muna, Marcus and Scott, 1969; Lewis *et al.*, 1969; Rohmsdahl and Cox, 1970).

Four of these 5 groups of workers described antibody to melanoma cells in the sera of approximately 60% of melanoma patients and in 10–20% of control sera. While similar final results have been obtained there have been marked differences in techniques, antibody titres obtained and even in the intracellular location of the fluorescence.

This report describes a study of sera from 57 melanoma patients with varying degrees of tumour spread and 39 normal sera using immunofluorescent techniques with 6 melanoma cell lines.

### MATERIALS AND METHODS

The cell lines used were all derived from secondary melanomata, either subcutaneous deposits or metastatic lymph nodes. Four of the cell lines (MM96, MM127, MM138 and MM170) have been described in detail elsewhere (Whitehead and Little, 1972). The other 2 cell lines (MM181 and MM182) were used at early passage levels and have not been studied in depth. Details of the cell lines used are given in Table I.

TABLE I.—*Details of Melanoma Cell Lines Used*

| Designation | Origin | Pigmentation | Passage level |
|---|---|---|---|
| MM 96 | Lymph node | + | 40–60 |
| MM 127 | Nodule | − | 20–30 |
| MM 138 | Nodule | + | 20–30 |
| MM 170 | Lymph node | + | 15–20 |
| MM 181 | Nodule | − | < 10 |
| MM 182 | Lymph node | + | < 10 |

The cells grew as monolayers and were removed by scraping or by treatment with versene (0·02%). The cells were suspended in RPMI 1640 + 2% foetal calf serum and were dropped on to acid cleaned slides and allowed to air dry. The smears were fixed either in cold acetone for 20 min or in liquid nitrogen–isopentane (Lewis *et al.*, 1969) and again air dried before use. Cells from apparently normal fibroblast cultures obtained from primary melanoma cultures, or from foetal skin, were treated in the same way and used as controls.

Sera were obtained from melanoma patients on the day of operation. Thirty-nine control sera were obtained from laboratory staff, patients having naevi or warts removed and in the case of the New Guinea sera, were from an Epstein–Barr virus study (J. H. Pope, personal communication). For the purpose of this study patients were considered as "cured" if they were tumour free 3 years or more after tumour removal.

Where possible, the primary melanomata were classified by one pathologist according to the criteria of Clark (1967) and J. H. Little (personal communication). However, a few sera from melanoma patients were received from other sources and in these cases the patient's tumour was not classified in this way.

Sera were stored at −20°C and diluted 1/6 for use. Sera were incubated with smears of the cells at room temperature for 30 min. The smears were washed 3 times in phosphate buffered saline (pH 7·2) and incubated with FITC-conjugated antihuman gamma globulin (Baltimore Biological Laboratories, Maryland) for 30 min at room temperature. After washing again, the smears were partially dried and mounted in a drop of 90% glycerol (pH 8·5). The smears were examined using a Leitz Orthoplan microscope with a dark field condenser and a HBO 200 light source fitted with a UG1 transmission filter and a K430 barrier filter. Sera were considered to be positive when more than 10% of the cells on the smear showed cytoplasmic fluorescence.

The conjugate was absorbed twice with acetone dried bovine liver powder (once at 37°C for 1 hour and once at 4°C overnight), and then twice with packed MM96 cells in a similar way, before use. The conjugate was diluted 1/15 and stored at −70°C until used. A conjugate control was included in each test but on no occasion did the conjugate alone stain the melanoma cells. The conjugate was shown to have anti IgG and anti IgM activity by immunodiffusion.

All sera giving definite cytoplasmic fluorescence with melanoma cells were also tested against foetal and adult skin fibroblasts and for antinuclear factor and smooth muscle antibody. Any sera positive in any of these tests were discarded.

### RESULTS

Preliminary experiments comparing fixation in cold acetone with fixation by freezing in liquid nitrogen–isopentane yielded similar results. Acetone fixation was used in subsequent studies as cells adhered better using this method. Fluorescence was localized in the cytoplasm and was normally diffuse in distribution.

The results obtained are summarized in Table II. The cell lines varied greatly in reactivity with the different groups of sera, but this variation was not related to either the pigmentation of the cells or length of time in culture. The overall incidence of fluorescence in tests with sera from melanoma patients was 32% whereas 17% of tests with sera from control patients gave positive results ($P < 0{\cdot}01$, $\chi^2$ test).

When the immunofluorescence reactions are tabulated in relation to the degree of spread of the patients' tumour

TABLE II.—*Summary of Immunofluorescence Reactions of Sera with Melanoma Cell Lines*

| Sera | Melanoma cell lines 96 | 127 | 138 | 170 | 181 | 182 | Total no. of tests |
|---|---|---|---|---|---|---|---|
| Normal (Caucasian) | 3/29* (10%) | 6/34 (18%) | 6/32 (19%) | 9/39 (23%) | 2/10 (20%) | 0/12 | 26/156 (17%) |
| Melanoma | 14/49 (29%) | 23/48† (48%) | 12/49 (24%) | 23/57 (40%) | 10/45 (22%) | 13/47 (28%) | 95/295† (32%) |
| Normal (New Guinea) | 4/6 (66%) | NT | 4/6 (66%) | 4/6 (66%) | NT | NT | 12/18† (66%) |

* Proportion of sera reacting.
† Significantly different ($P < 0{\cdot}01$) from value for normal sera using $\chi^2$ test.
NT Not tested.

TABLE III.—*Immunofluorescence Reactions of Sera from Patients with Melanoma*

| Tumour stage | Cell lines 96 | 127 | 138 | 170 | 181 | 182 | Total no. of tests |
|---|---|---|---|---|---|---|---|
| Primary | 4/25* (16%) | 6/20 (30%) | 3/25 (12%) | 10/27 (37%) | 3/23 (13%) | 3/24 (12%) | 29/144 (21%) |
| Secondary | 8/18 (44%) | 10/20 (50%) | 7/19 (37%) | 8/22 (36%) | 5/16 (31%) | 6/15 (40%) | 44/110† (40%) |
| " Cured " | 2/6 (33%) | 7/8 (87%) | 2/5 (40%) | 5/8 (62%) | 2/6 (33%) | 4/8 (50%) | 22/41† (54%) |
| Total no. of tests | 14/49 (29%) | 23/48 (48%) | 12/49 (24%) | 23/57 (40%) | 10/45 (22%) | 13/47 (28%) | 95/295 (32%) |

* Proportion of sera reacting.
† Significantly different ($P < 0 \cdot 01$) from value for sera from primary patients for using $\chi^2$ test.

TABLE IV.—*Immunofluorescence Reactions of Sera from Patients with Primary Melanoma*

| Depth of invasion (Clark, 1967) | Cell line 96 | 127 | 138 | 170 | 181 | 182 | Total no. of tests |
|---|---|---|---|---|---|---|---|
| Epidermis or upper dermis (Stage 1 and 2) | 0/5* | NT | 0/5 | 0/5 | 0/4 | 0/4 | 0/23 |
| Lower dermis (Stage 3A and B) | 4/13 | 5/14 | 3/16 | 7/17 | 3/17 | 3/17 | 25/94 |

* Proportion of sera reacting.
NT Not tested.

(Table III), it is apparent that sera from patients with secondary melanoma reacted more frequently than did sera from patients with primary melanoma ($P < 0 \cdot 01$, $\chi^2$ test). Sera from patients considered to be " cured " of their tumour were found to react more frequently than any other group of sera.

Analysis of results of sera from patients with primary melanoma which had been staged for depth of invasion according to Clark's classification (1967) showed that no serum from any patient whose tumour was restricted to the epidermis or upper dermis (stage 1 or 2) reacted with any of the melanoma cell lines (Table IV). However, sera from patients whose tumour had invaded the lower dermis were positive in up to 40% (7/17 against MM170) of cases.

The results may be examined in a different way: of 22 sera from patients with primary melanoma tested against all 6 cell lines, 12 reacted with at least one cell line (Table V). Similarly, of 16 sera from patients with secondary melanoma tested against all cell lines, 13 reacted against at least one cell line (Table V). However, the cell lines showed very little cross-reactivity with sera from melanoma patients. No primary serum reacted with all 6 cell lines and only 3/22 sera reacted with 3 or more of the cell lines. Sera from patients with secondary melanoma showed more cross-reactivity and 7/16 sera reacted with at least 3 of the cell lines.

TABLE V.—*Cross-reactivity of Sera from Patients with Melanoma against the Six Melanoma Cell Lines*

| Number of cell lines reacted with | Primary melanoma sera reacting | Secondary melanoma sera reacting |
|---|---|---|
| 6 | 0 | 1 |
| 5 | 0 | 2 |
| 4 | 1 | 2 |
| 3 | 2 | 2 |
| 2 | 5 | 0 |
| 1 | 4 | 6 |
| 0 | 10 | 3 |
| Total | 22 | 16 |
| Total positive | 12 (55%) | 13 (81%) |

Six of the secondary melanoma sera that were tested against all 6 melanomata reacted with one cell line only. Three of these reacted with MM96, 2 with MM127 and one with MM182. Of the 2 sera reacting with 4 cell lines, both reacted with MM127, MM170 and MM182, one also reacted with MM138 and the other with MM181. Sixteen secondary sera were tested fully and there were a total of 36 reactions. MM127 and MM182 were reacted 8 times, MM170 7 times, MM96 6 times, MM138 4 times and MM181 3 times. These results are an indication of the diversity of reactions found.

### DISCUSSION

This report describes a much lower incidence of antibody to melanoma than that found by most other authors using immunofluorescent techniques. However, the findings are in accordance with those of Nairn *et al.* (1972), who have dealt in detail with the possible reasons for these discrepancies. Although early reports tended to agree as to the proportion of positive sera, the techniques and methods of assessment differed. For instance, Morton *et al.* (1968) described both nuclear and cytoplasmic fluorescence and used nuclear fluorescence as the indicator of reactivity to 3 of their melanomata whereas most other workers have disregarded nuclear fluorescence. The titre of the antibody detected in the serum has varied from 1/2 (Muna *et al.*, 1969) to 1/125 (Oettgen *et al.*, 1968). Other variations are seen in the methods used to fix the cells and even the source of the cells themselves.

It was decided to exclude any sera containing antinuclear factor or smooth muscle antibody, or reacting with adult or foetal fibroblasts, from this study. Most previous authors have not adequately excluded these factors and doubts have been expressed as to the specificity of the reactions described. It was realized that by eliminating these sera, some sera also showing anti-melanoma reactivity might be excluded.

The results indicate that melanoma cells in culture do contain an antigen which reacts with sera from some melanoma patients. However, the cell lines which are most reactive with melanoma sera are also most reactive with normal sera. Similar results are also seen in the reports of Morton *et al.* (1968) and Rohmsdahl and Cox (1970). Morton *et al.* reported that their most reactive melanoma (measured by both nuclear and cytoplasmic fluorescence) reacted with 76% of sera from melanoma patients and 43% of control sera. Similarly, Rohmsdahl found that a melanoma which reacted with 89% of sera (measured by cytoplasmic fluorescence) from melanoma patients also reacted with 43% of normal sera (Table VI). The high reactivity of

TABLE VI.—*Comparison of Results of Cell Line showing Most Reaction with Control Sera*

| Sera | Morton *et al.* (1968) | Rohmsdahl *et al.* (1970) | Whitehead |
|---|---|---|---|
| | | % Positive | |
| Melanoma | 76 | 89 | 40 |
| Normal | 43 | 43 | 23 |

sera from normal New Guineans is also difficult to explain, but is similar to a previous report from Lewis *et al.* (1969) who found that 21/25 normal negro sera reacted with melanoma cells by immunofluorescence.

Hellström *et al.* (1973) have recently reported that lymphocytes from healthy North American negroes are cytotoxic to cultured melanoma cells. They also found that sera from these people are able to " unblock " sera from patients with melanoma. This suggests that there is an antibody in the serum of normal Negro people that can combine with circulating antigen in the serum of a patient with melanoma. These findings, taken together, indicate the presence of an antibody capable of reacting with melanoma antigens in the blood of normal dark skinned people and may explain why melanomata are less common in dark skinned people.

The implication is that there is an antigen in melanoma cells, either new or unmasked, which reacts with sera from some patients with melanoma. However, the fact that approximately 20% of normal sera also react with melanoma cells argues against this new antigen being melanoma specific.

The fact that the proportion of sera positive can be described as 21% (Table III) or 55% (Table V) for sera from primary melanomata and either 40% (Table III) or 81% (Table V) for sera from secondary melanomata, indicates the dangers inherent in analysing data such as these. No serum from a patient with primary melanoma reacted with all 6 melanoma cell lines, and only 43% of sera from patients with secondary melanomata reacted with 3 or more of the cell lines. These findings provide no evidence for the presence of the common antigen in melanoma cells suggested previously by Morton *et al.* (1968) and Lewis *et al.* (1969).

However, it is possible that the tumour specific antigen was lost or greatly diminished at an early stage of the *in vitro* culture or that relatively non-antigenic cell clones were selected by the tissue culture conditions used.

I wish to thank Dr J. H. Little and his colleagues of the Queensland Melanoma Project for obtaining the blood from melanoma patients. I also wish to thank Dr R. L. Doherty and Dr J. H. Pope for their initial guidance and continuing encouragement and advice and Miss Marie Steen and Mr P. Byth for their able technical assistance.

### REFERENCES

CLARK, W. H. (1967) A Classification of Malignant Melanoma in Man Correlated with Histogenesis and Biologic Behavior. *Adv. Biol. Skin*, **8**, 621.

HELLSTRÖM, I., HELLSTRÖM, K. E., SJÖGREN, H. O. & WARNER, G. A. (1973) Destruction of Cultivated Melanoma Cells by Lymphocytes from Healthy Black (North American) Donors. *Int. J. Cancer*, **11**, 116.

LEWIS, M. G. (1967) Possible Immunological Factors in Human Malignant Melanoma in Uganda. *Lancet*, ii, 921.

LEWIS, M. G., IKONOPISOV, R. L., NAIRN, R. C., PHILLIPS, T. M., HAMILTON FAIRLEY, G., BODENHAM, D. C. & ALEXANDER, P. (1969) Tumour-specific Antibodies in Human Malignant Melanoma and their Relationship to the Extent of Disease. *Br. med. J.*, iii, 547.

MORTON, D. L., MALMGREN, R. A., HOLMES, E. C. & KETCHEM, A. S. (1968) Demonstration of Antibodies against Human Malignant Melanoma by Immunofluorescence. *Surgery, St Louis*, **64**, 233.

MUNA, N. M., MARCUS, S. & SMART, C. (1969) Detection by Immunofluorescence of Antibodies Specific for Human Malignant Melanoma Cells. *Cancer, N.Y.*, **23**, 88.

NAIRN, R. C., NIND, A. P. P., GULI, E. P. G., DAVIES, D. J., LITTLE, J. H., DAVIS, N. C. & WHITEHEAD, R. H. (1972) Anti-tumour Reactivity in Patients with Malignant Melanoma. *Med. J. Aust.*, **1**, 397.

OETTGEN, H. F., AOKI, T., OLD, L. J., BOYSE, E. A., DE HARVEN, E. & MILLS, G. M. (1968) Suspension Culture of a Pigment Producing Cell Line Derived from a Human Malignant Melanoma. *J. natn. Cancer Inst.*, **41**, 827.

ROHMSDAHL, M. M. & COX, I. S. (1970) Human Malignant Melanoma Antibodies Demonstrated by Immunofluorescence. *Archs Surg.*, **100**, 491.

WHITEHEAD, R. H. & LITTLE, J. H. (1972) *Tissue Culture Studies in Malignant Melanoma.* In Proc. VIIIth. Internat. Pigment Cell Congr. Basle: Karger A.G. In the press.